# Biochemical and morphological responses to post-hepatectomy liver failure in rats

**DOI:** 10.1038/s41598-023-40736-y

**Published:** 2023-08-19

**Authors:** Andrea Lund, Kasper Jarlhelt Andersen, Michelle Meier, Marie Ingemann Pedersen, Anders Riegels Knudsen, Jakob Kirkegård, Frank Viborg Mortensen, Jens Randel Nyengaard

**Affiliations:** 1 Department of Surgery, Section for Upper Gastrointestinal and Hepato-Pancreato-Biliary Surgery, Aarhus University Hospital, Palle Juul-Jensens Boulevard 35, 8200 Aarhus N, Denmark; 2 Department of Clinical Medicine, Aarhus University, Aarhus, Denmark; 3 Core Center for Molecular Morphology, Section for Stereology and Microscopy, Department of Clinical Medicine, Aarhus University, Aarhus, Denmark; 4 Department of Pathology, Aarhus University Hospital, Aarhus, Denmark

**Keywords:** Hepatocytes, Cell division, Cell growth, Hepatocytes, Liver diseases

## Abstract

The upper limit for partial hepatectomy (PH) in rats is 90%, which is associated with an increased risk of post-hepatectomy liver failure (PHLF), correlating with high mortality. Sixty-eight rats were randomized to 90% PH, sham operation, or no surgery. Further block randomization was performed to determine the time of euthanasia, whether 12, 24, or 48 h after surgery. A general distress score (GDS) was calculated to distinguish between rats with reversible (GDS < 10) and irreversible PHLF (GDS ≥ 10). At euthanasia, the liver remnant and blood were collected. Liver-specific biochemistry and regeneration ratio were measured. Hepatocyte proliferation and volume were estimated using stereological methods. All rats subjected to 90% experienced biochemical PHLF. The biochemical and morphological liver responses did not differ between the groups until 48 h after surgery. At 48 h, liver regeneration and function were significantly improved in survivors. The peak mean regeneration ratio was 15% for rats with irreversible PHLF compared to 26% for rats with reversible PHLF. The 90% PH rat model was associated with PHLF and high mortality. Irreversible PHLF was characterized by impaired liver regeneration capacity and an insufficient ability to metabolize ammonia.

## Introduction

Partial hepatectomy (PH) is an important treatment for both primary and secondary liver malignancies^1,2^. The liver has a unique regeneration ability, and in healthy humans, up to 75% of the liver volume can be surgically removed without detrimental effects on liver function^3,4^. Substantially exceeding this threshold increases the risk of post-hepatectomy liver failure (PHLF), which is a severe complication with a high mortality rate^5,6^.

Biochemically, PHLF is characterized by hyperbilirubinemia and impaired coagulation^7^. No cure exists for PHLF, which means that early detection and organ support are the only available treatment options^6^.

Investigating the pathophysiological processes involved in PHLF is essential for identifying new biological markers of this severe condition. This may help improve outcomes after extensive PH and guide intervention efforts to avoid and treat PHLF. Systematic randomized studies of the pathophysiology underlying PHLF in humans are impossible for ethical reasons. The experimental rat model of PH can be used to bypass this issue because it is homogeneous and ideal for both PHLF and regeneration studies.

Only 5–10% of the initial liver volume is required to maintain vital liver functions in rats^8–10^, which is lower than the 25–30% required for humans, so the 90% PH experimental rat model approaches the PHLF threshold^11–13^. This model simulates the critical period of the minimal-size future liver remnant, during which it is determined whether PH is followed by reversible PHLF and regeneration, or irreversible PHLF and death.

For rats with reversible PHLF, we previously found 90% PH to be followed by hepatocyte hypertrophy and proliferation after 24 and 78 h, respectively^14^. In this study, we aimed to investigate and compare reversible and irreversible PHLF in rats regarding functional liver capacity and regeneration ability.

## Materials and methods

### Ethical approval

The experimental protocol was approved by the Danish Animal Research Committee, Copenhagen, Denmark (licence number: 2021-15-0201-00978). Animals received care in accordance with the US National Institutes of Health’s *Guide for the Care and Use of Laboratory Animals*^15^. The study is reported in accordance with ARRIVE guidelines^16^.

Healthy, 7–8-week-old male Wistar rats (Janvier Labs, Le Genest-Saint-Isle, France) with a mean preoperative weight of 270 g (range: 240–300 g) were housed in standard animal laboratories with a temperature maintained at 23 °C, an artificial 12-h light–dark cycle, and free access to food and water. The animals were kept in these facilities until the end of the experiment.

### Experimental design

**Figure 1:**
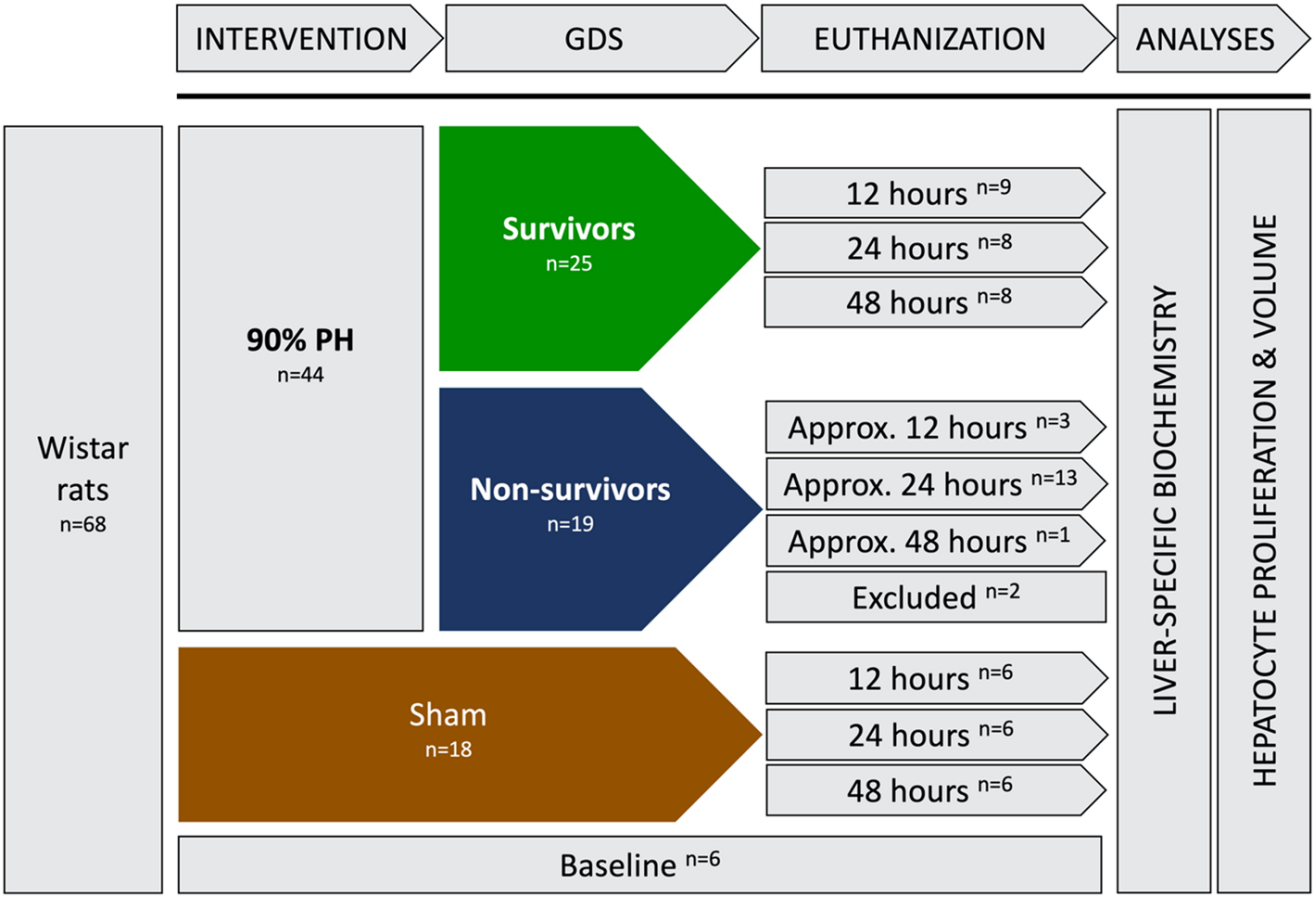
Study design including a total of 68 male Wistar rats randomized according to intervention and time of euthanasia. Two animals were excluded in the analyses: 1 died unexpectedly and 1 due to vena cava compression.

Sixty-eight male Wistar rats were randomized in blocks of two to either 90% PH (n = 44) or sham operation (n = 18) (Fig. 1). Each group was block randomized into three subgroups for euthanasia at 12 h (n = 10), 24 h (n = 14), or 48 h (n = 20) after surgery.

Animals presenting with a general distress score (GDS)^17^ ≥ 10 during day or GDS ≥ 6 at midnight were euthanised. Our previous publication provides a comprehensive description of the GDS evaluation^18^. Animals euthanised before planned evaluation were allocated to the “non-survivors” group and the subgroup closest to the actual time of euthanasia. Animals with a GDS < 10 at the planned time of euthanasia were allocated to the “survivors” group. A baseline group (n = 6) not undergoing laparotomy was also included.

### Surgical procedure and tissue sampling

We previously described the surgical and anaestetic procedures in detail^18^. In brief, the surgical procedure was performed with inhalation anesthesia, using a mixture of oxygen (0.3 L/min), nitrous oxide (0.15 L/min), and sevoflurane (5%). 90% PH was performed by resecting the left lateral lobe, mediane lobe, right superior lobe, and right inferior lobe, leaving only the anterior caudate lobe and posterior caudate lobe intact. Sham-operated animals were subjected to laparotomy without PH.

The animals were observed continuously for the first three hours after surgery and then at least four times per day using a GDS. Two independent observers allocated a GDS value to each animal. Early euthanasia was performed if the GDS was ≥ 10 at any time during the day or ≥ 6 at midnight. Animals that died unexpectedly were autopsied to establish the cause of death.

At 12, 24, or 48 h, the animals were anesthetised again. Blood samples were collected from the heart by cannulation, and the liver remnant, consisting of the anterior and posterior caudate lobes, was harvested. Euthanasia was performed by cervical dislocation under anaesthesia.

### Biochemical analyses

Blood samples were processed, snap-frozen in liquid nitrogen and stored at -80 °C until analysis. Alanine aminotransferase (ALT), alkaline phosphatase, haptoglobin, bilirubin, albumin, ammonia, creatinine, sodium, potassium and phosphate were measured using an immunoassay and clinical chemistry analyser (Siemens Healthcare Diagnostics Inc., Erlangen, Germany). The prothrombin-proconvertin ratio (PP ratio) was measured using Sysmex CS-2100i (Sysmex Corporation, Kobe, Japan), an automated blood coagulation analyser.

### Liver weight and regeneration ratio

The pre-operatively estimated liver weight was calculated from the resected liver weight:

$$Estimated\, liver\, weight(preoperatively)= \frac{Resected\, liver\, weight}{Size\, of\, PH}\times 100$$

The change in liver weight was considered the regeneration ratio:

$$Regeneration\, ratio=\frac{\frac{Liver\, weight\left(euthanization\right)}{Body\, weight(euthanization)}}{\frac{Estimated\, liver\, weight\left(preoperatively\right)}{Body\, weight(preoperatively)}} \times 100$$

### Stereology

#### Tissue preparation

The posterior caudate lobe was fixed in phosphate-buffered formalin for 24–48 h and cut into 2-mm thick parallel slices using a tissue slicer. Tissue slices were placed with the same side up and embedded in paraffin. From each paraffin-embedded block, three 3-µm thick sections were cut, providing systematic, uniformly random sampling sections for immunostaining and further analysis^19^.

#### Immunohistochemistry

Immunohistochemical staining of the serial sections was performed using an automated slide stainer (Benchmark Ultra; Ventana Medical Systems, Roche, Basel, Switzerland). All sections were deparaffinised and boiled in Ventana CC1 buffer (Ventana Medical Systems, Roche, Basel, Switzerland), pH 8, for heat-induced epitope retrieval. The sections were incubated for 32 min with two different antibodies. Ready-to-use monoclonal mouse anti-rat Ki-67 specific antibody (clone 30–9; Ventana Medical Systems, Roche, Basel, Switzerland) diluted at 1:20 was used as a proliferation marker, and anti-beta-catenin antibody (clone β-Catenin-1; Agilent Technologies, Santa Clara, CA, U.S.) diluted at 1:100 was used as a hepatocyte cell surface marker. The sections were counterstained with haematoxylin. An OptiView DAB Detection Kit (Roche, Basel, Switzerland) and UltraView Universal Alkaline Phosphatase Red Detection Kit (Roche, Basel, Switzerland) were then used for visualization of the bound antibodies.

#### Quantification

An investigator blinded to the section treatments analysed the sections using an Olympus BX50 microscope, modified for stereology using a motorized stage (Märzhäuser Wetzlar MFD, Wetzlar, Germany) and a digital camera (Olympus DP73, Olympus, Tokyo, Japan) connected to a computer running newCAST version 2020.08.4.9377 software (Visiopharm, Hørsholm, Denmark). The same investigator analysed all the sections.

Three thin serial sections were used to estimate the total number of Ki-67-positive-stained hepatocyte nuclei profiles per area of liver tissue. Microscopy was performed using a 60 × oil objective lens (NA 1.30). A 2-dimensional (2D) unbiased counting frame (65 × 45 μm^2^) was used to sample hepatocyte nuclei profiles, in, on average, 100 systematically uniformly and randomly selected fields of view per animal. Positive staining was defined as a counterstained red oval hepatocyte nucleus. In the 48-h survivor group, approximately 150 positive stained hepatocyte nuclei were counted per animal. Corners of the counting frame formed 4 test points. For every counting frame, the number of test points overlapping the liver tissue was counted.

**Figure 2:**
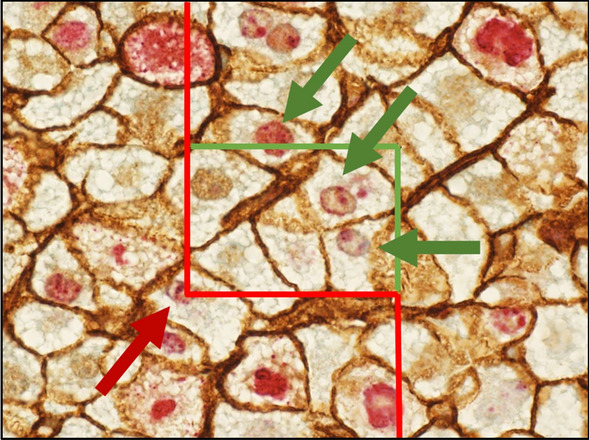
Ki-67 and beta-catenin-stained section from a 48-h survivor rat. Counting frames are displayed; green lines are inclusion lines and red lines are exclusion lines. The universal counting rule states that a positive hepatocyte profile is counted if the nucleus is significantly stained and if it is entirely within the counting frame, or if it touches an inclusion line and does not touch an exclusion line (green arrows). Positive nuclei touching an exclusion line are not counted (red arrow)^19^.

Total count of Ki-67-positive hepatocyte nuclei per area (N_A_) was calculated using the following formula:

$${\mathrm{N}}_{\mathrm{A}}=\frac{\sum \mathrm{Q}}{\frac{\mathrm{a}}{\mathrm{p}} x \sum \mathrm{P}}$$

where ΣQ is the total count of Ki-67-positive hepatocyte nuclei profiles using the 2D unbiased counting frame in three sections, a/p is the area of the unbiased counting frame divided by four, and ΣP is the sum of the test points overlapping the liver tissue in the sections. The counting rules are illustrated in Fig. 2.

**Figure 3:**
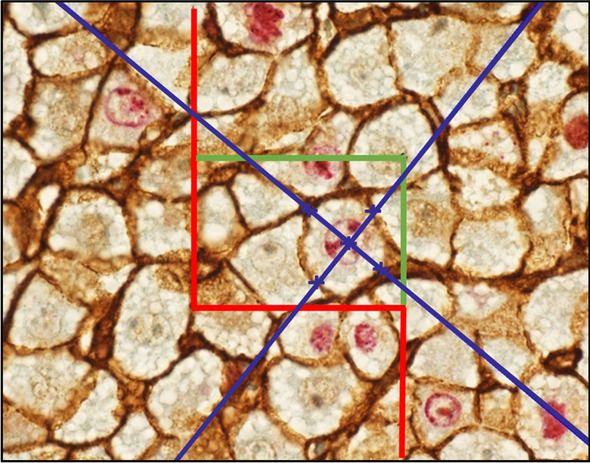
Ki-67 and beta-catenin-stained section from a 48-h survivor rat. Hepatocyte volumes are estimated using the principles of the 3D isotropic nucleator on a 3 µm-thick section. Counting frames are displayed as red exclusion lines and green inclusion lines. The universal sampling rule is described in Fig. 2. In this study, the objects of interest were hepatocytes, both Ki-67-positive and -negative, with a clear brown beta-catenin-stained cell membrane. The sampling unit was the hepatocyte nuclear profile. The software then generated two systematically random sampled test lines running through the nucleus of the hepatocyte. Intercept lengths of the cell membrane were marked. By using the distance along the two isotropic lines radiating from the nucleus to the boundary of the cell membrane, the software estimated the volume of the hepatocytes.

Hepatocyte volumes were estimated on hepatocytes with a beta-catenin-stained brown cell membrane using a 3D isotropic nucleator (Fig. 3). The nucleator was applied to each of the sampled hepatocyte profiles. The software calculated the volume of each sampled hepatocyte. For each section, mean hepatocyte volume was calculated before ratio-of-sums for the three serial sections was measured.

### Statistical analyses

All variables were tested for normality using histograms and QQ-plots. When normality was violated, data were log-transformed to ensure normality, analyzed, and then back-transformed. The groups were compared using an analysis of variance, whereas an unpaired *t*-test was used for pairwise comparisons. Mean values are shown with their 95% confidence intervals (CIs). *P*-values of < 0.05 were considered significant. To evaluate intra-observer variance, the number of positive hepatocyte nuclei was first counted for all animals. Hereafter, the investigator randomly selected 8 sections from the 90% PH groups and counted the number of positive hepatocyte nuclei once again. The agreement between the first and second counts was calculated using intra-class correlation coefficients (two-way mixed model). For inter-observer variation in GDS measurements, Cohen’s weighted kappa was calculated for two raters. All statistical analyses were performed using Stata version 17.0 (StataCorp LLC, College Station, TX, U.S.).

## Results

### Mortality and morbidity after 90% PH

During the experiment, two of 68 animals were excluded: One died during surgery due to vena cava compression, and one died unexpectedly overnight. All animals in the study were autopsied after euthanasia. Except for the one animal with vena cava compression, none of the autopsied animals revealed identifiable causes of mortality or morbidity.

The mean survival time of non-survivors with a GDS ≥ 10 was 27 h (95% CI 23–30). Mortality was 60% (95% CI 38–80%) for animals randomized to euthanasia 48 h after PH. Inter-observer agreement in GDS measurements was high (kappa: 0.98; 95% CI 0.97–1.00).

### Liver-specific biochemistry

The biochemical parameters are shown in Table 1. ALT, bilirubin, alkaline phosphatase, and ammonia were elevated at all times after PH compared to baseline values and sham treated animals. PP ratio dropped dramatically after PH and remained low compared with baseline values. Haptoglobin was markedly suppressed in the 90% PH groups compared with the sham treatment groups. No difference was found between survivors and non-survivors in liver-specific biochemistry for the first 24 h after PH. After 48 h, ALT and ammonia started decreasing for survivors.

**Table 1:** Raw dataset of liver-specific biochemistry. Mean values with 95% CI. Lower CI is truncated at 0.

	Survivors			Non-survivors			Sham			Baseline
	12	24	48	12	24	48	12	24	48	
Bilirubin µmol/L	18 (16;21)	44 (23;64)	45 (31;60)	25 (18;31)	46 (39;54)	45 -	< 3 (< 3; < 3)	< 3 (< 3; < 3)	< 3 (< 3; < 3)	< 3 (< 3; < 3)
PP ratio	.08 (.04;.12)	.18 (0;.38)	.09 (.06;.12)	.05 (.05;.05)	.10 (.02;.18)	0.07 -	.26 (.09;.44)	.41 (.35;.48)	.39 (.33;.46)	.36 (.19;.52)
ALT U/L	2018 (1627; 2409)	2126 (1640; 2613)	1313 (810; 1817)	2698 (0; 12,666)	2516 (1747; 3284)	2401 -	52 (40;65)	54 (44;64)	49 (37.2; 60.7)	43 (26;60)
Ammonia µmol/L	418 (358; 479)	561 (490; 633)	300 (210; 389)	455 (0; 1281)	640 (551; 728)	*-*	43.67 (0;87)	15 -	34.5 (3;66)	47 (9;85)
Alkaline Phosphatase U/L	607 (526; 688)	656 (558; 755)	841 (729; 953)	715 (0; 1445)	795 (692; 897)	830 -	240 (212; 269)	224 (203; 245)	202 (137; 269)	270 (199; 340)
Haptoglobin g/L	< .01 (01;.01)	< .01 (01;.01)	< .01 (01;.01)	< .01 (01;.01)	< .01 (01;.01)	< .01 -	.285 (.22;.35)	0.44 -	0.55 -	0.08 (0;.27)

### Body weight and regeneration ratio

**Figure 4:**
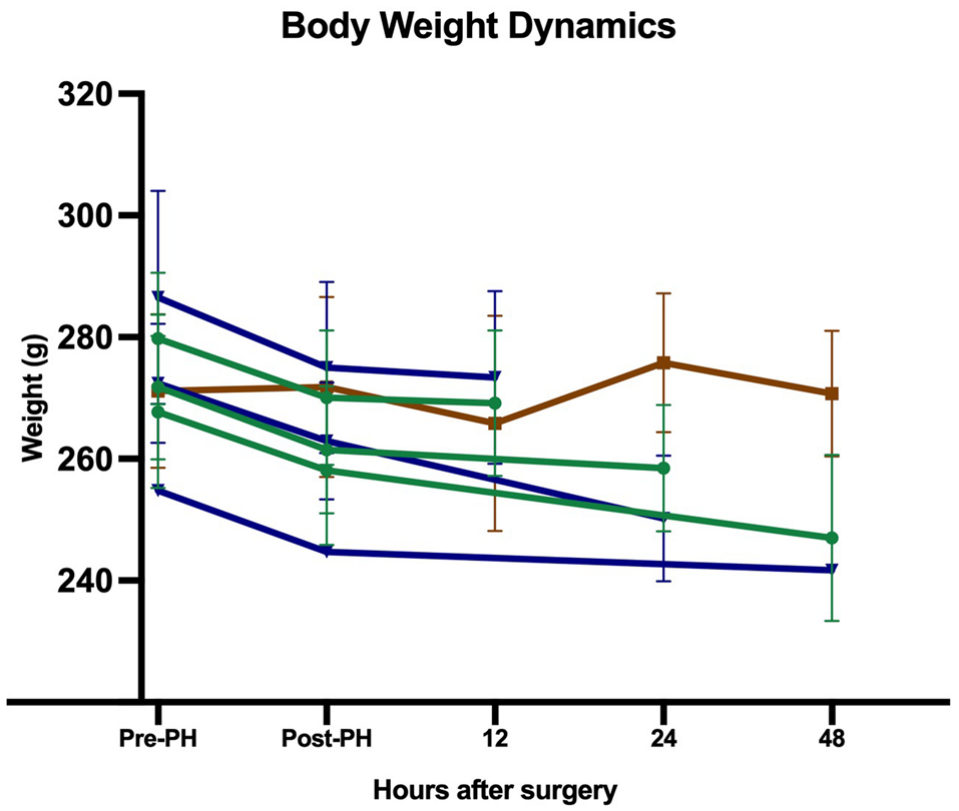
Mean body weight from pre-surgery to euthanasia. Through the study, no significant body weight differences between survivors and non-survivors were found. Green lines: survivor subgroups, blue lines: non-survivor subgroups, brown line: sham.

**Figure 5:**
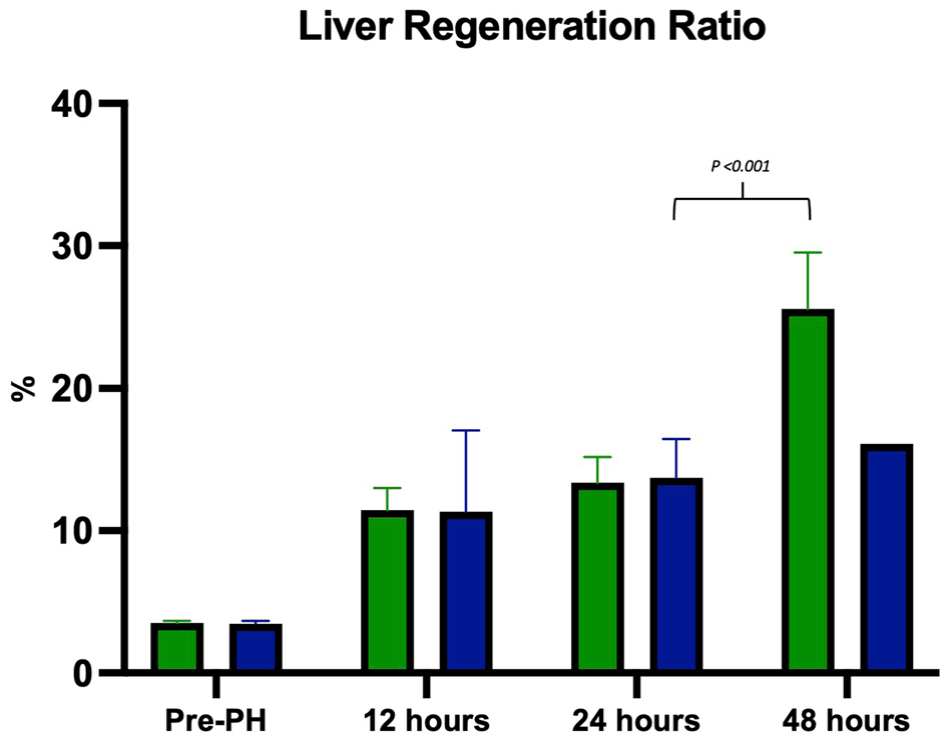
Mean regeneration ratio by euthanasia group. Within 24 h after PH, no significant difference was found between survivors and non-survivors. At 48 h, regeneration ratio increased markedly in survivors. Green line: survivors, blue line: non-survivors.

For all 90% PH animals, body weight loss was observed throughout the study period. As shown in Fig. 4, no significant difference in body weights of the survivors and non-survivors was found^18^. The mean resected liver weights of the survivors and non-survivors were 8.6 g (95% CI 8.2–9.0) and 8.5 g (95% CI 8.0–9.0), respectively. There was significant increase in the regeneration ratio of the 48-h survivors compared with that of all the other subgroups (Fig. 5). Raw mean values are presented in Table 2.

**Table 2:** Raw data including ratios of sums for hepatocyte proliferation, volume of proliferating and non-proliferating hepatocytes, and regeneration ratio. Mean values with 95% CI. Lower CI is truncated at 0.

	Survivors			Non-survivors			Sham			Baseline
	12	24	48	12	24	48	12	24	48	
Proliferating hepatocytes per mm	87 (33;141)	26 (0;69)	790 (493; 1088)	73 (0;347)	17 (8;27)	6 -	45 (10;80)	7 (0;17)	3 (0;9)	21 (0;45)
Volume of proliferating hepatocytes (µm^3^)	6833 (4736; 8930)	6833 (4736; 8929)	10,602 (8516; 12,689)	5431 (2734; 8127)	5608 (4176; 7039)	4673 -	4478 (3928; 5027)	4796 (4463; 5130)	5493 (4380; 6607)	4702 (4084; 5319)
Volume of non-proliferating hepatocytes (µm^3^)	4488 (4075; 4900)	4598 (3993; 5204)	8777 (6652; 10,902)	3858 (1384; 6331)	5498 (4805; 6192)	5932 -	4572 (4349; 4794)	4210 (3680; 4741)	4323 (3533; 5043)	4186 (3304; 5068)
Regeneration ratio (%)	12 (10;13)	13 (12;15)	26 (22;30)	11 (6;17)	14 (11;16)	16 -	NA	NA	NA	NA

### Hepatocyte proliferation

**Figure 6:**
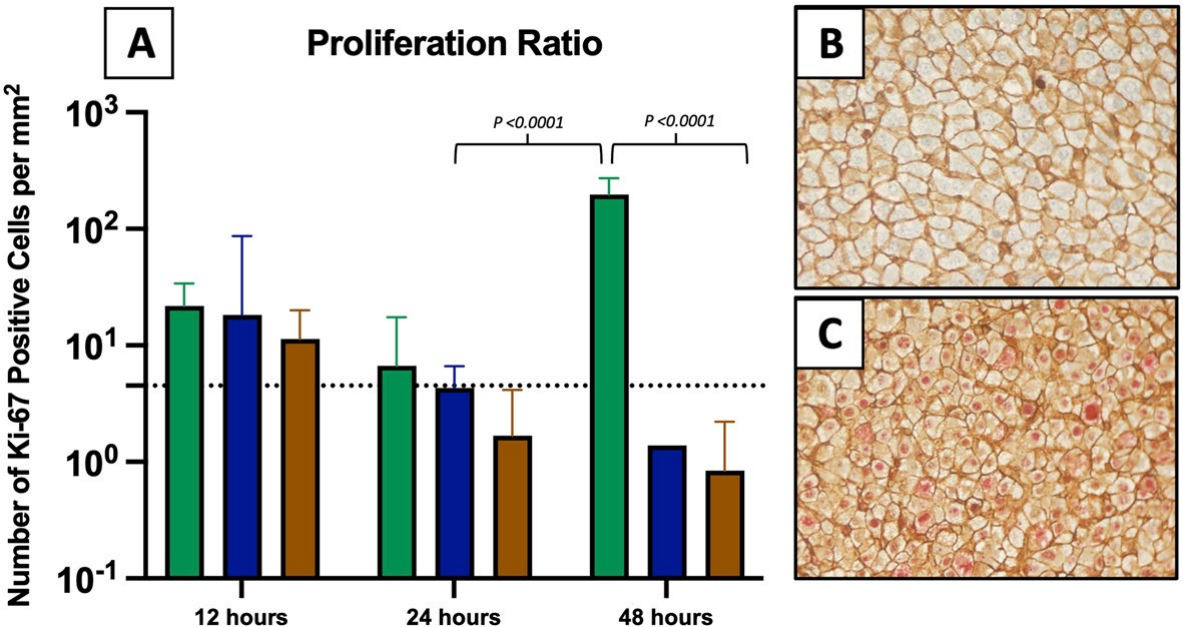
Proliferation of hepatocytes by euthanasia group. (**A**) The logarithm to hepatocyte proliferation ratio by euthanasia group. Within 24 h after PH, no significant difference in proliferating hepatocytes was found between survivors and non-survivors. At 48 h, an accelerated proliferation response was found for survivors. Green line: survivor, blue line: non-survivor, brown line: sham, and dotted line: baseline. (**B** + **C**) Ki-67 and beta-catenin-stained sections from a 24-h non-survivor (**B**) and a 48-h survivor rat (**C**) using a 20 × objective lens. No counterstained red nuclei are seen for the non-survivor compared to multiple Ki-67-positive nuclei for the survivor.

**Figure 7:**
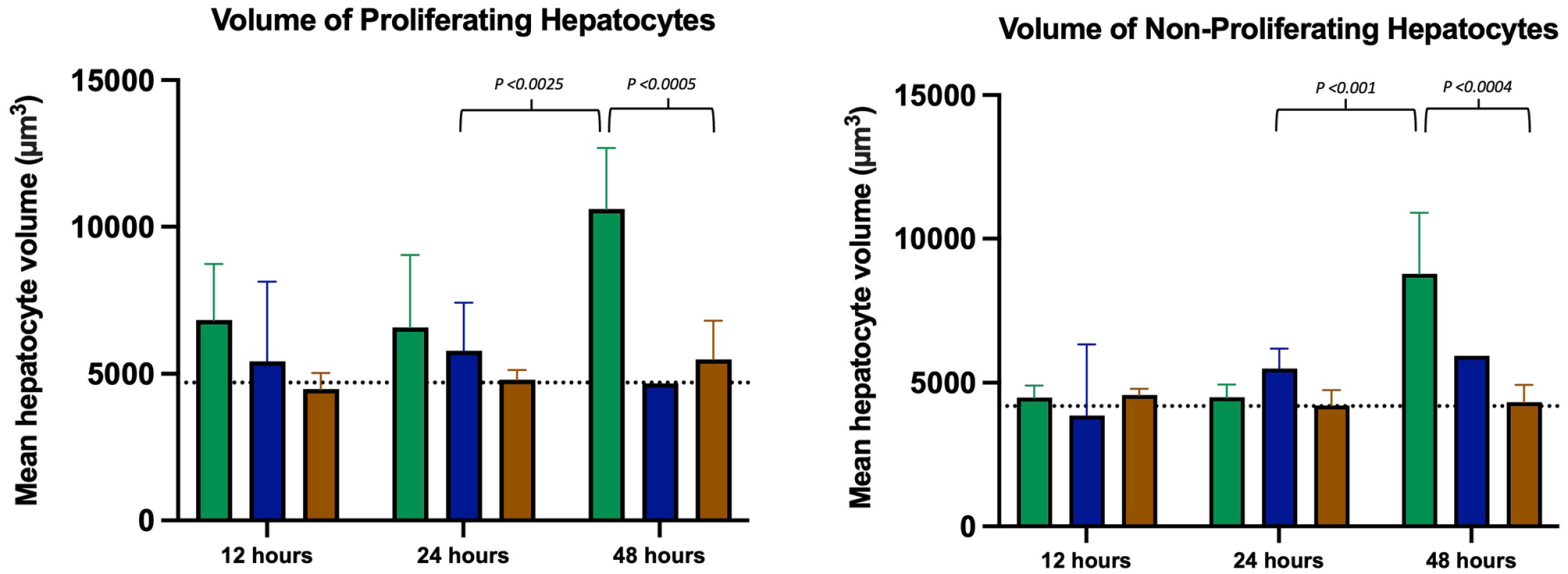
Volume of proliferating and non-proliferating hepatocytes by euthanasia group. Within 24 h after PH, no significant differences in hepatocyte volumes were found between survivors and non-survivors. At 48 h, the volume of both proliferating and non-proliferating hepatocytes increased markedly in survivors. Green: survivor, blue: non-survivor, brown: sham, and dotted line: baseline.

Within 24 h after surgery, there was no significant increase in hepatocyte proliferation in the PH group compared with the baseline and sham-operated groups. The survivors and non-survivors did not proliferate differently within this period. At 48 h, there was a significant increase in Ki-67-positive hepatocytes in the 48-h survivor group compared with the other subgroups (Figs. 6 and 7). The intra-class correlation coefficient, as a measure of intra-observer agreement in hepatocyte nuclei counts, was 0.97 (95% CI 0.92–1.00).

### Hepatocyte volume

There was no significant increase in hepatocyte volume in the PH rats compared with the baseline and sham-operated rats until 48 h after surgery. At 48 h, there was a significant increase in volume for both proliferating and non-proliferating hepatocytes in the survivors compared to the non-survivors.

## Discussion

In the present study of a 90% PH model in rats, all animals developed PHLF. The survivors and non-survivors did not differ in either biochemical or morphological liver responses within 24 h after PH. After 48 h, liver regeneration was significantly accelerated, and liver function was significantly improved in the survivors as expressed by increased hepatocyte hypertrophy and proliferation and a decline in serum ammonia levels.

PHLF is often characterized by post-operatively acquired deterioration in the ability to maintain synthetic, excretory, and detoxifying liver functions, thereby compromising homeostasis^7,20^. In our study, we defined PHLF as low PP ratio and high bilirubin level, as the latter is widely considered a marker of liver failure^7^. In all the animals that underwent 90% PH, PP ratio was markedly suppressed, and bilirubin was increased. Low PP values are markers of impaired synthetic function, and elevated bilirubin levels can likely be explained by decreased hepatocellular uptake, decreased conjugation or decreased biliary excretion^21^. We found no significant differences between the survivors and non-survivors in any biochemical parameters measured until 48 h after PH, at which point ALT and ammonia decreased towards baseline levels, suggesting improvements in the liver function of the survivors. Elevated ALT levels signify liver injury. The decrease in ALT levels 48 h after PH pointed to liver recovery. Hyperammonia after PH suppresses ureagenesis, resulting in a reduced ability of the liver remnant to eliminate neurotoxic ammonia. The resulting metabolic challenges may be partly due to the loss of hepatocytes and partly due to alterations in hepatic perfusion^22,23^. Decreasing ammonia levels signify improved liver detoxification, which could, at least partly, explain the reversal of PHLF after PH.

Similar to the biochemical measurements, we found no significant increase in either hepatocyte hypertrophy or hyperplasia until 48 h after PH. We then observed a significant regeneration response among the survivors with a doubling in the mean hepatocyte volume and a substantial increase in the mean number of Ki-67-positive hepatocyte nuclei. From 12 h after PH, we found a tendency towards increased volumes of proliferating hepatocytes in the survivors. In comparison, the volumes of hepatocytes in the non-survivors remained the same as those in the sham-operated animals throughout the study.

In line with the findings of other studies^14,24^, our results indicate that in the early phase of PHLF, hepatocyte hypertrophy is preserved, whereas proliferation is transiently suppressed. Based on observations suggesting that hepatic lipid and amino acid accumulation are beneficial for hepatocyte proliferation after liver resections^24–26^, it seems plausible that the hypertrophic response might act as a primer for proliferation. It is possible that a volume threshold exists for hepatocyte proliferation although this is of course speculative. Nevertheless, hyperplasia was not observed in terms of liver regeneration after the induction of irreversible PHLF.

In a previous study on rats with reversible PHLF, we evaluated liver function and liver morphology on days 1, 3 and 5 after 90% PH^11^. We found that liver function was impaired (defined by low PP ratio and high bilirubin level) until post-operative day 5 but that it began to normalize from day 3. We detected the greatest mean hepatocyte volume on day 1 and a peak in the number of proliferating cells on day 3. Liver function was improved on day 3 compared to what we found on day 2 in the present study. Our combined findings suggest that rat liver functional capacity improves following liver regeneration. Thus, in the early phase of PHLF, the liver prioritizes regeneration over maintenance of body homeostasis.

We used unbiased stereological methods to assess hepatocyte proliferation and volume. For estimates of the volume of individual cells in sections in the absence of 3D reconstruction, two requirements must be met. First, the cells must be disector sampled either by an optical or a physical disector. Second, the sections must be ‘isotropic’ or ‘vertical’^27^. In terms of cell volume measurements, Møller et al. showed that the results obtained using a thin section and a 2D counting frame were almost equivalent to those obtained by dissector sampling, when cell volume was measured on sampled nucleoli profiles^28^. As nucleoli cannot be detected in hepatocytes, we used hepatocyte nuclei as sampling units, sampling nuclei in 2D counting frames in thin serial sections. Using nuclei to sample hepatocytes is probably not as valid a measure as using nucleoli, as it could result in underestimation of hepatocyte volume. However, the potential underestimate seems minor. We do not have ‘isotropic’ or ‘vertical’ sections in our study. However, we assume that hepatocytes on a global scale, assessed in several sections, are isotropic. Furthermore, we assume equal shrinkage in all paraffin sections.

A simple quantitative GDS, which we have previously evaluated, was used to distinguish rats with reversible PHLF from rats with irreversible PHLF^18^. In our study, there was no identifiable cause of death at autopsy in any of the animals, and GDS ≥ 10 seemed to be a valid non-invasive marker of irreversible PHLF. One of the parameters evaluated in calculating the GDS is body weight. Body weight is not only a measure of water and food intake but also a reliable marker of acute stress in rats^29^. All animals that underwent PH showed a pronounced and persistent decline in body weight after surgery compared to the sham-operated animals. No significant difference in body weight dynamics were found between the survivors and non-survivors. This finding indicates that the procedure alone elicits a major physiological stress response, independent of whether PHLF is reversible or irreversible. Our findings are in agreement with our previous results in a study where body weight remained low during the first 5 days after 90% PH in rats^11^.

There is limited evidence of the pathophysiology of PHLF after 90% PH in rats. Previous studies found that PHLF-related mortality occurred within 72 h after surgery^11,12^, with 70% of the animals dying within 24 h^12^. This finding is in agreement with that of the present study, in which 60% of PHLF-related mortality occurred within 24 h after surgery. In our previous study, we detected no difference in GDS of the survivors and non-survivors until 18 h after PHLF^18^. Therefore, we do not know whether the PHLF would have turned out for the 12-h survivor group, if they had not been euthanised. It is also unknown whether PHLF would have proved fatal in some of the 24-h survivors at a later stage, as the mean time for PHLF-related mortality was 27 h after PH. In our study, all the euthanasias performed within 12 h after surgery were due to either surgical complications (*n* = 1) or a GDS of > 6 (*n* = 2). Therefore, these animals were no included in the analysis, as their deaths were not due to PHLF-related complications. Consequently, the 24-h non-survivors seem to be the most representative group of PHLF-related mortality.

Our study has some important strengths that improve its validity. First, we used stereological methods to assess adaptive changes in hepatocyte morphology. Compared with qualitative or semi-quantitative histological approaches, stereological methods make it possible to obtain accurate quantitative information on 3D structures from 2D samples in a randomized and unbiased manner^30,31^. Second, to minimize bias due to potential variances between litters, all the animals were randomized according to the intervention and euthanasia time. Third, two observers evaluated all the animals to mitigate inaccuracies in GDS evaluations, and hepatocyte nuclei counts were done twice to check for intra-observer variance.

The observational gap of 6 h at night is a limitation of our study. For ethical reasons, we decided to euthanise animals with a GDS ≥ 6 at midnight. However, as only one animal died for no known reason overnight, and two animals were euthanised at midnight due to a GDS of 6, the lack of observations during the night is of limited concern. A priori, we expected mortality to be evenly distributed within the first 48 h after surgery. To gain sufficient statistical power for all subgroups, at least eight animals in each group would have been optimal. However, it was not possible to have eight animals in all the non-survivor subgroups, as only two animals had a GDS ≥ 10 outside the time frame of the 24-h subgroup.

In conclusion, our research revealed that reversal of PHLF in rats is dependent on the ability of the liver remnant to regenerate while maintaining both body homeostasis and the metabolism of ammonia. Our study highlights that rats suffering from fatal PHLF lack the ability to sufficiently regenerate their liver, as demonstrated by the absence of hepatocyte proliferation. Additionally, we provide biochemical evidence indicating that rats dying of PHLF exhibit insufficient ammonia metabolism. The findings of this study are important for future research aimed at understanding the pathophysiology of PHLF after extensive PH.

## Data availability

All data generated or analyzed during this study are included in this published article.
