# Publisher Correction: Biochemical and morphological responses to post-hepatectomy liver failure in rats

**DOI:** 10.1038/s41598-024-73048-w

**Published:** 2024-09-26

**Authors:** Andrea Lund, Kasper Jarlhelt Andersen, Michelle Meier, Marie Ingemann Pedersen, Anders Riegels Knudsen, Jakob Kirkegård, Frank Viborg Mortensen, Jens Randel Nyengaard

**Affiliations:** 1 Department of Surgery, Section for Upper Gastrointestinal and Hepato‑Pancreato‑Biliary Surgery, Aarhus University Hospital, Palle Juul‑Jensens Boulevard 35, 8200 Aarhus N, Denmark; 2 Department of Clinical Medicine, Aarhus University, Aarhus, Denmark; 3 Core Center for Molecular Morphology, Section for Stereology and Microscopy, Department of Clinical Medicine, Aarhus University, Aarhus, Denmark; 4 Department of Pathology, Aarhus University Hospital, Aarhus, Denmark

Correction to: *Scientific Reports*10.1038/s41598-023-40736-y, published online 19 August 2023

The original version of this Article contained errors in the References.

In the Materials and methods section, under the subheading ‘Ethical approval’, Reference 16 was omitted. Consequently,

“The study is reported in accordance with ARRIVE guidelines (16)”.

now reads:

“The study is reported in accordance with ARRIVE guidelines^16^.”

As a result, the subsequent References have been renumbered.

The errors have now been corrected in the PDF version of the Article; the HTML version was correct from the time of publication.

